# Interventions That Use Highly Visual Social Media Platforms to Tackle Unhealthy Body Image in Adolescents and Young Adults: Systematic Review of Randomized Controlled Trials and Quasi-Experimental Studies

**DOI:** 10.2196/80141

**Published:** 2026-02-09

**Authors:** Anthony Copez-Lonzoy, Janina Bazalar-Palacios, Nikol Mayo-Puchoc, Gustavo Calderon de la Cruz, Liliana Cruz-Ausejo, Evelyn Goicochea-Ríos, Juan Carlos Bazo-Alvarez

**Affiliations:** 1 Bibliometrics Unit Universidad San Ignacio de Loyola Lima, Lima Province Peru; 2 Research Unit PSYCOPERU Peruvian Institute for Psychological and Psychosocial Research Ancash, Chimbote Peru; 3 Facultad de Ciencias de la Salud Universidad Tecnológica del Perú Lima, Lima Province Peru; 4 Mental Health Unit Instituto Peruano de Orientación Psicológica Lima Peru; 5 School of Psychology Peruvian University of Applied Sciences Lima Peru; 6 School Medicine National University of San Marcos Lima Peru; 7 Grupo MedFam Escuela de Medicina Universidad César Vallejo Trujillo Peru; 8 Research Department of Primary Care and Population Health University College London London United Kingdom; 9 Grupo MedFam Escuela de Psicología Universidad Cesar Vallejo Trujillo Peru

**Keywords:** adolescent, body image, digital intervention, microinterventions, social media, systematic review, young adult

## Abstract

**Background:**

Highly visual social media (HVSM) platforms such as Facebook (Meta Platforms, Inc), Instagram (Meta Platforms, Inc), TikTok (ByteDance Ltd), and Snapchat (Snap Inc) have become central to the digital lives of adolescents and young adults. While these platforms have been linked to body dissatisfaction, they are also increasingly used as vehicles for health promotion. However, the evidence on interventions delivered through HVSM to address body image issues remains fragmented.

**Objective:**

This review aimed to synthesize available evidence on interventions using HVSM platforms to reduce negative body image in adolescents and young adults.

**Methods:**

We conducted a systematic search across 5 electronic databases (Scopus, MEDLINE, APA Psynet, Embase, and Web of Science) for studies published between January 2012 and October 2025. Eligible studies included experimental or quasi-experimental designs evaluating the effect of an HVSM-based intervention on body image outcomes in individuals aged 13 to 35 years. Risk of bias was assessed using the Risk of Bias Tool 2.0 (Cochrane) and was conducted independently by 2 researchers.

**Results:**

Eight studies met the inclusion criteria with 4975 participants (2612 in intervention groups and 2363 in control groups). Most studies were conducted in high-income countries and had predominantly female participants. The interventions varied widely in format, duration, and theoretical basis. Microinterventions, brief interactive strategies such as gamified chatbots or short videos, were the most common and had moderate effects. Stimulus-based interventions using content with a positive body image or that did not focus on appearance were also identified, achieving moderate effects (η_p_²<.07), as well as combined approaches that integrated digital and face-to-face components to reduce negative body image (*P*<.001). The use and functionality of interventions using social media platforms were also compared by gender.

**Conclusions:**

Body image management platforms offer an emerging avenue for implementing body image interventions in adolescents and young adults. While current evidence suggests modest benefits, the high heterogeneity among presentation formats and the variability in duration make comparisons between these studies difficult. This review synthesizes social media–delivered interventions for body image disturbance, going beyond broader digital approaches centered on websites or apps. It identifies cross-platform, putative mechanisms of action and common intervention formats, highlighting the potential of brief interventions for scalable reach and user empowerment via content curation. These findings define targets for optimization and underscore the need for platform safeguards and supportive policy and regulatory frameworks to enable safe real-world implementation, particularly for adolescents.

## Introduction

Body image (BI) is a person’s perception of their physical appearance and the potential positive or negative thoughts about self-worth based on that perception [1]. The problems associated with this negative perception of BI can affect various levels of health, such as eating disorders, risky behaviors, and excessive dieting [2]. This generates a public health problem affecting diverse age and gender populations [3]. Among the various mental health problems, negative BI remains a major concern for young people, especially adolescent girls [4]. According to research, between 20% and 40% of adolescent girls experience severe body dissatisfaction, which often persists or worsens during adulthood [4,5]. However, this prevalence of negative BI can reach 35% to 81% in adolescent girls and 16% to 55% in adolescent boys in low- and middle-income countries [5].

There is a strong correlation between BI and self-esteem. Adolescents and young adults frequently develop erroneous self-perceptions as a result of unrealistic physique ideals promoted by social media platforms [6]. According to research, excessive use of social media increases body dissatisfaction, promotes unfavorable comparisons, and lowers self-esteem [7]. This combination of low self-esteem and distorted BI increases the risk of developing eating disorders such as anorexia and bulimia nervosa [8]. Systematic reviews show that body dissatisfaction predicts the beginning of eating disorders and is linked to long-term mental health difficulties such as sadness and anxiety [9].

Adolescents and young adults are among the most active social media users, with more than 90% reporting frequent use of at least one site such as Instagram, TikTok, or Snapchat (Snap Inc) [10]. Globally, social media users reach 60% of the population, with adolescents and young adults frequently having accounts on various platforms and switching between them depending on age, cultural influences, and geographic location [11]. These platforms are intentionally designed to increase user engagement with features such as tailored content feeds, targeted notifications, and tools for modifying and sharing multimedia material [12]. Highly visual social media (HVSM) platforms, such as Instagram and TikTok, have transformed the digital environment by emphasizing photos and videos, making visual appeal an important driver of user retention [13,14]. These systems’ algorithms continuously adjust content delivery to match user preferences, increasing interactivity and encouraging long-term engagement [15]. This dynamic produces a digital environment that captivates adolescents and young adults while also influencing their actions and beliefs, especially those connected to self-image and mental health [16].

Though highly visually appealing social media (HVSM) platforms are designed to enhance user engagement and dependency, they also have great potential as tools for mental health screening and intervention among teenagers and young adults. Recent research shows how feasible and successful digital mental health interventions through social media (eg, including HVSM platforms) [17-20]. These types of interventions overcome the accessibility and affordability limitations of traditional mental health services [21]. They can be administered at scale, are cost-effective to implement, and are easy to train, improving access to mental health services for individuals, thereby reducing the workload of health personnel [22].

Despite these developments, there are still important gaps in methodically summarizing treatments aimed at poor BI using HVSM. For example, little data exist on which particular platforms best handle BI issues and the special characteristics of interventions catered to these platforms [23]. Nevertheless, some alternatives use affordances of HVSM, such as specific algorithms, visual material, and interaction, to assess and minimize negative concerns about BI among adolescents and young adults. Nonetheless, further investigation is required to find best practices for using HVSM-based therapies, so that they are accessible, culturally relevant, and able to generate quantifiable changes in BI and general mental health [24,25], ultimately leading to the creation of creative and scalable solutions for urgent problems.

We aim to summarize evidence on the effectiveness of interventions that use HVSM platforms to tackle unhealthy BI in adolescents and young adults.

## Methods

### Overview

The systematic review was conducted according to the PRISMA (Preferred Reporting Items for Systematic Reviews and Meta-Analyses) and PRISMA-S (Preferred Reporting Items for Systematic Reviews and Meta-Analyses Search Extension) guidelines [26-28] (Multimedia Appendices 1 and 2). The study protocol was published in open access [29].

### Eligibility Criteria

Only studies included (1) adolescent and/or young adult populations (only groups with an age range from 13 to 35 years were included), (2) randomized clinical trials or quasi-experimental studies that evaluated BI interventions, (3) studies that fully or partially used social media as an intervention (eg, Facebook [Meta Platforms, Inc], Instagram [Meta Platforms, Inc], and TikTok [ByteDance Ltd)], or other interactive social media), and (4) studies that evaluated outcomes directly related to BI (eg, body dissatisfaction, body satisfaction, self-esteem, disordered eating, and appearance satisfaction). Studies that meet any of these criteria were excluded (1) studies that are not original journal articles (letters, book chapters, systematic reviews, and meta-analyses), (2) studies that cannot be considered interventions (eg, without pre or postevaluation and random assignment), or that are comparison conditions (eg, stimuli only), (3) studies that include a higher age range than established (eg, university students more than 35 years old at the time of the intervention), (4) studies that use social media to build the intervention, and (5) studies before 2012 (the first study that used social media as part of the intervention dates back to 2012) [30].

### Information Sources

The initial search was conducted during 2024 and the final search was conducted up to October 10, 2025. Five databases were consulted (Scopus, Embase, Web of Science, Medline, and PsycINFO) in their entirety (ie, only studies that are listed in the databases). Only the information published in the studies was taken into account. We examined MeSH (Medical Subject Headings) terms for relevance and frequency of potentially included studies. We used validated filters to locate adolescent-based and young adult–based HVSM interventions where appropriate (eg, title, keyword, or abstract). AC-L and JB-P conducted citation tracking of initially included studies included in Scopus, identifying previously excluded studies and some of the studies included in the final review. Throughout the search process, standardized terms (eg, MeSH for MEDLINE) and Boolean operators (AND, OR) were applied to ensure a replicable process for each database.

### Search Strategy

The following search strategy was used, based on the literature on BI and social media interventions in adolescents and young adults. In addition, publication year filters were included (eg, pubyear >2011). We include search strategies on BI, Mahon and Seekis [31], and adapt them to the needs of this study. The search strategies were peer reviewed by 2 team members (ACL and JBP) and an external expert (senior information specialist) prior to execution. All identified fields (eg, social media AND intervention) were unified to generate the search strategy. BI terms (appearance OR “body anxiety” OR “body attitude” OR “body attitudes” OR “body checking” OR “body concern*” OR “body esteem” OR “body evaluation” OR “body satisfaction” OR “body dissatisfaction” OR “body image” OR “body functionality” OR “body surveillance” OR “body shame” OR “body positive” OR “body positivity” OR “body acceptance” OR “body pride” OR “body preoccupation” OR “body awareness” OR “body anxiety” OR “body appreciation” OR “body neutrality” OR “physical self-perception” OR “physical self-perception” OR “physical self-concept” OR “physical self-concept” OR “shape concern” OR “shape concerns” OR “weight concern*” OR “shape satisfaction” OR “shape dissatisfaction” OR interocep* OR “weight dissatisfaction” OR “weight satisfaction” OR “body ideal*” OR “appearance ideal*” OR muscularity OR “thin ideal” OR “physical appearance” OR muscular* OR “social physique anxiety” OR “self-objectification” OR self-objectification OR “objectified body consciousness” OR thinness OR “cognitive restraint” OR “body identity” OR “body schema” OR “body representation*” OR “self-esteem” OR “eating disorders”), social media terms (internet OR “social media” OR “social network site*” OR instagram OR facebook OR “tik tok” OR “TikTok” OR twitter OR reddit OR telegram OR whapsapp OR weibo OR snapchat OR youtube OR pinterest), population terms (Child* OR adolescent* OR young OR youth OR teen* OR college OR universit* OR “Young Adult*”) randomized controlled trial (RCT) and quasi-experimental terms (intervention* OR “controlled trial” OR “clinical trial” OR “major clinical trial” OR “quasi-experimental” OR “quasi experimental”).

We limited the results to studies from 2012 onward, as this year saw the proliferation of large-scale studies on social media (eg, Facebook, Instagram, TikTok, or other HVSMs) [13]. The search strategy for each database is in Zenodo [32] or refer to Multimedia Appendix 3.

### Selection Process and Data Collection

After eliminating duplicates, 2 researchers (ACL and JBP) independently assessed the titles and abstracts of all articles based on established criteria using Rayyan software (Qatar Computing Research Institute) [33]. We removed results that did not provide sufficient information from the full-text review. We repeated this process for the full-text phase. Data were extracted using a digital form predesigned by the research team. The form contains information on (1) general characteristics of the studies: first author, journal, country, study design (eg, RCT and quasi-experimental), participants or target population (Table 1); (2) social media: social media components and features used for the intervention or evaluation; (3) interventions: type of intervention, level of intervention (eg, country and hospital), duration of intervention, methodological details (eg, details of the design for the effectiveness evaluation, sample size, and instruments), effectiveness estimate and/or its estimation (eg, mean difference); and (4) assessment tools: construct or object to be assessed (eg, image, video, chatbot, or others), self-reports used one or more dimensions [34,35] (scales or inventories; Table 2).

**Table 1:** Description of intervention characteristics and highly visual social media platform features in 8 studies [24,25,36-41] to improve body image.a

Author (year)	Country	Body image outcomes	Social media platform	Affordances	Duration	Delivered by	Intervention description
Sampson et al [25] (2020)	United Kingdom	Body and facial dissatisfaction	Instagram	Photos	2 weeks	Researchers	They explored the assigned images on specific individual devices (eg, iPads), comparing the viewing of idealized content (eg, attractive smiles) and neutral images in Instagram users for 5 minutes.
Seekis et al [40] (2020)	Australia	Body dissatisfaction, drive for thinness, body appreciation, and self-compassion	Facebook	Facebook group	2 weeks	Researchers: workshop and Facebook group moderator	Attendance at 50-minute in-person workshops (6 workshops) held for 2 days, with an average of 12 participants each. These workshops are based on 6 strategies from the Mindful Self-Compassion manual, delivered as psychoeducation on self-compassion for body image. In addition, discussions are held in a private Facebook group (3 times per 2 weeks) to share experiences and apply what was learned in the workshop.
Fioravanti et al [38] (2023)	Italy	Body satisfaction, appearance comparison	Instagram	Images	28 days	Researchers	Researchers created Instagram profiles based on “body positivity,” “encompassing fitspiration,” and “neutral content” images, creating images for each profile type (approximately 112 images). Participants were randomly assigned to each group to follow their respective profiles. Content was delivered daily (1 post + 3 Instagram stories) for 28 days. In addition, participants received 2 messages per day asking questions about the content they received on Instagram.
Garbett et al [41] (2023)	Indonesia	Body satisfaction, attitudes toward appearance	Facebook and Instagram	Video	30 minutes	Self-guided	Self-guided web-based activities on 6 sequential videos (~ 5 minutes each) on body image issues related to Indonesian youth, complemented by social media activities.
Pilot et al [39] (2023)	United States	Body dissatisfaction	Instagram	Images	Not clear	Researchers	Progressive viewing of 20 pairs of fitspiration images on Instagram contrasted with reading and generating a list of 12 personal values ​​and identifying the most important value.
Lewis-Smith et al [37] (2023)	United States	Body satisfaction, acceptance of appearance diversity and appearance-related internalized racism	YouTube	Video	5 minutes	Researchers	A theoretically grounded microintervention in the form of a dramatic miniseries (first episode only) aimed at improving body satisfaction, internalized appearance-related racism, and acceptance of appearance diversity (specifically, acceptance of larger-bodied people of Black people) among Black and non-Black adolescents (American Indian and Alaska Native, Asian, Native Hawaiian and Other Pacific Islands or White) in the United States.
Matheson et al [24] (2023)	Brazil	Body satisfaction, positive and negative affect	Facebook	Chatbot	3 days	Avatar	A chatbot operated through Facebook Messenger that provided microinterventions for improving body image and mental well-being based on psychoeducational techniques, interactive activities, and coping strategies.
Fardouly et al [36] (2023)	Australia	Body dissatisfaction and appreciation, self-objectification, appearance comparison tendency, and body activism	Facebook	Facebook group	14 days	Social media	Microintervention created in private Facebook groups was based on the exposure of body-positive content. Two different conditions were administered (positive and neutral body awareness). Participants were dosed with posts of content relevant to each condition for 14 consecutive days (3 times a day).

^a^Affordances = set of tools, characteristics, and options of social media platforms for interaction, creation, management, and sharing between users (eg, photos, private or open groups, text, video, etc).

**Table 2:** Essential characteristics and key findings from randomized controlled trials on the effectiveness of social media interventions to improve body image^a^.

Author (year)	Sample size	Age	Study design	Follow-up	Comparison group	Body images measures	Key findings	Did the intervention achieve its goals?
Sampson et al [25] (2020)	132	Mean 20.50 (2.21), range 18-30 years	Randomized controlled trial	N/A^b^	Yes, images of neutral nature	BSS^c^; FSS^d^; SACS^e^; SDI^f^	Exposure to “ideal” facial images on social media decreases facial satisfaction (*P*<.001).	Partially
Seekis et al [40] (2020)	76	Mean 18.04 (0.90), range 17-21 years	Cluster randomized trial	1-3 months	Yes, waitlist	Subscale EDI^g^; SAAS^h^; UPACS^i^; BAS-2^j^; SCS-SF^k^	The combined strategy (workshop + Facebook group) based on self-compassion reduced body dissatisfaction (β=–.74; *P*<.001).	Yes
Garbett et al [41] (2023)	1847	Intervention mean 16.94 (SD 1.40) years; control: mean 17 (SD 1.41), range 15-19 years	Randomized controlled trial	1 month	Yes, waitlist	BESAA^l^; SATAQ-3^m^; PANAS-C^n^	Warna-Warni Waktu is an effective eHealth intervention to reduce body dissatisfaction among adolescent girls and young women (*P*=.005, η^2^_p_=0.05).	Yes
Lewis-Smith et al [37] (2023)	686	Mean 15.72 (SD 1.60), range 13-18 years	Parallel randomized controlled trial	N/A	Yes, non–body image–related video	VAS^o^; subscale IROS^p^	No changes in body satisfaction were observed. The changes identified were only in conditions of racism and acceptance of diversity (*P*<.001; Cohen *d*>0.17).	No
Matheson et al [24] (2023)	1715	Range 13-18 years (52.54% girls)	Randomized controlled trial	1 week; 1 month	Yes, assessment-only control	BESAA; PANAS; BISES^q^	Body self-esteem identified improvements in positive (Cohen *d*=0.13; *P*=.02) and negative (Cohen *d*=0.11; *P*=.047) appearance.	Yes
Fardouly et al [36] (2023)	159	Mean 20.92 (SD 2.21), 18-25 years	Parallel randomized controlled trial	4 weeks	Yes, assessment-only control	Subscale EDI; BAS-2; PANAS-SF^r^; SOBBS^s^; PACS^t^; SAM^u^ modified	Body dissatisfaction was reduced in the body positive Facebook group condition (*P*=.03; η_p_^2^=0.05) with small effect.	Yes
Pilot et al [39] (2023)	238	Mean 19.89 (SD 1.25), 18-22 years	Randomized controlled trial	N/A	Yes, travel images	Subscale EDEQ^v^; WREQ^w^; VAS	The intervention based on Values ​​Affirmation + Fitspiration does not show significant changes in body image (*P*=.73) or in negative mood (*P*=.60) compared to the control.	No

^a^Relevant results for BI = The information was assessed by the team as “no” (no favorable results for body image), “partially” (favorable results for some body image outcomes), and “yes” (favorable results for body image outcomes).

^b^N/A: not applicable.

^c^BSS: Body Satisfaction Scale.

^d^FSS: Facial Satisfaction Scale.

^e^SACS: State Appearance Comparison Scale.

^f^SDI: Self-Discrepancy Index.

^g^EDI-3: Eating Disorder Inventory.

^h^SAAS: Social Appearance Anxiety Scale.

^i^UPACS: Upward Physical Appearance Comparison Scale.

^j^BAS-2: Body Appreciation Scale-2.

^k^SCS-SF: Self-Compassion Scale–Short-Form.

^l^BESAA: Body Esteem Scale for Adolescents and Adults.

^m^SATAQ-3 Sociocultural Attitudes Towards Appearance Questionnaire.

^n^PANAS-C: Positive and Negative Affect Schedule for Children.

^o^VAS: Visual Analogue Scales.

^p^IROS: Internalized Racial Oppression Scale (Alteration of Physical Appearance and Hair Change subscales).

^q^BISES: Body Image Self-Efficacy Scale.

^r^PANAS-SF: Positive and Negative Affect Scale-Short Form.

^s^SOBBS: Self-Objectification Beliefs and Behaviors Scale.

^t^PACS: Physical Appearance Comparison Scale.

^u^SAM: modified version of the social activism measure.

^v^EDEQ: Eating Disorder Examination Questionnaire (weight or shape subscales).

^w^WREQ: Weight-related eating questionnaire.

### Risk of Bias

Methodological quality was assessed using the Risk of Bias Tool (RoB 2; Cochrane Collaboration) established by the Cochrane System of Interventions Assessment Handbook [42]. This tool evaluates 5 domains: the randomization process, deviations from planned interventions, missing data, outcome measurement, and selection of reported outcomes. We classified the judgment rating for each domain as “yes,” “probably yes,” “no,” “probably no,” and “unreported.” Furthermore, the combination of the 5 domains provides an overall score that classifies the risk as “low,” “high,” or “some concerns.” Two reviewers (AC-L and JB-P) conducted independent assessments of the studies included in this paper. We resolved any disagreements through discussions with a third reviewer (JCB-A).

### Synthesis Methods

The information was systematically synthesized into tables containing descriptive information on the selected results. Tables were generated for the descriptive results and separated by specific design type (eg, RCT or quasi-experimental). Figures were generated based on the specific characteristics of the included studies and the methodological quality assessment by domain and overall (risk of bias). The information was grouped taking into account 4 intervention conditions that use social media as microinterventions, stimulus comparison, combined interventions, and self-guided videos. A narrative synthesis was chosen due to two critical conditions that prevented a meta-analysis: (1) the wide heterogeneity previously identified in the studies such as the different populations (eg, adolescents, young adults, or both), social media platforms (Facebook, Instagram, YouTube, or combined), affordances (ie, photos, images, videos, Facebook groups, post or chatbots), duration of interventions (eg, 5 minutes, 3 days, and up to 14 days) and follow-up (1 week to 1 month) and (2) the small number of studies included (*k*=3) per condition assessed (eg, body satisfaction or dissatisfaction) would not allow a subgroup analysis to understand the possible source of heterogeneity that could affect clinical decision-making as mentioned in the Cochrane guide [43].

### Ethical Considerations

The research ethics committee from Universidad Cesar Vallejo, with a code of 100-CEI-EPM-UCV-2022, reviewed and approved this study. No additional permissions were required as the data were based on articles published in different databases accessible to the authors (AC-L, JB-P, and JCB-A).

## Results

### Study Selection

**Figure 1:**
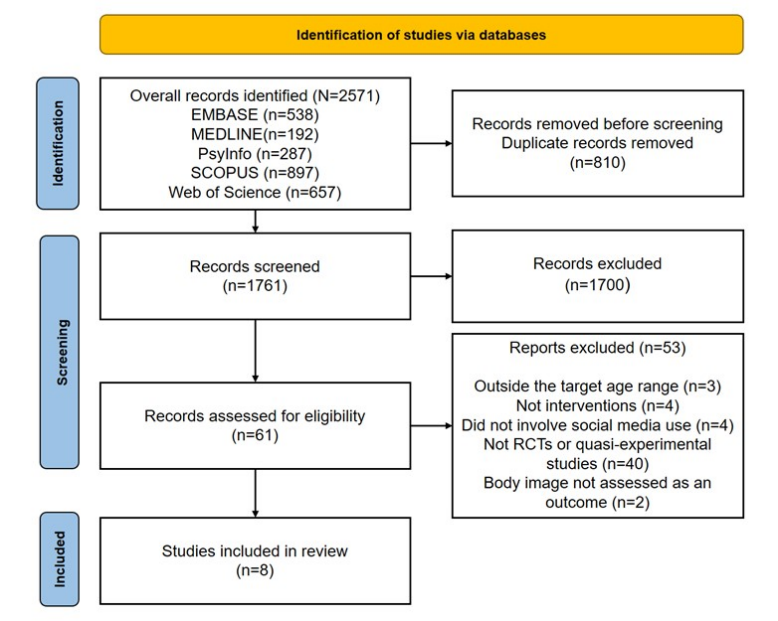
PRISMA (Preferred Reporting Items for Systematic Review and Meta-Analysis) flow diagram describing the literature review process. RCT: randomized controlled trial.

A total of 2571 references were identified across all databases reviewed in the literature search. After identifying duplicates, 1761 articles were retained for review by title and abstract. Sixty-one full-text articles were included. Of these 61 articles, 53 were excluded, the following exclusion characteristics were outside the age range (3/53, 5.7%) were not an intervention (4/53, 7.5%), did not use social media (4/53, 7.5%) did not have RCTs or quasi-experimental (40/53, 75.5%) and the outcome was not related to BI (2/53, 3.8%) leaving 8 articles for this review (Figure 1).

### Descriptive Characteristics of the Studies

Table 1 shows an overview of the studies identified by social media year, country, and social media use. All identified studies were published between 2020 and 2023, with a majority based in the American Continent (Brazil and the United States). The majority of studies were conducted on female participants (6/8, 75%), and only 2 studies were conducted for mixed-gender groups [24,25]. The main platform for intervention (partial or total) was Facebook (4/8, 50%). The intervention duration ranged from 5 minutes to 28 days, and in most cases, the intervention was delivered by the researchers (6/8, 75%). The characteristics of the identified interventions were microinterventions to improve body satisfaction (n=3) [24,36,37], combined interventions (workshop + online group; n=1) [40], stimulus-based interventions with the presentation of socially “ideal” and “attractive” images (n=3) [25,38,39], and a self-guided model based on sequential videos to combat appearance-related pressures (n=1) [41].

### Characteristics of the Interventions

**Figure 2:**
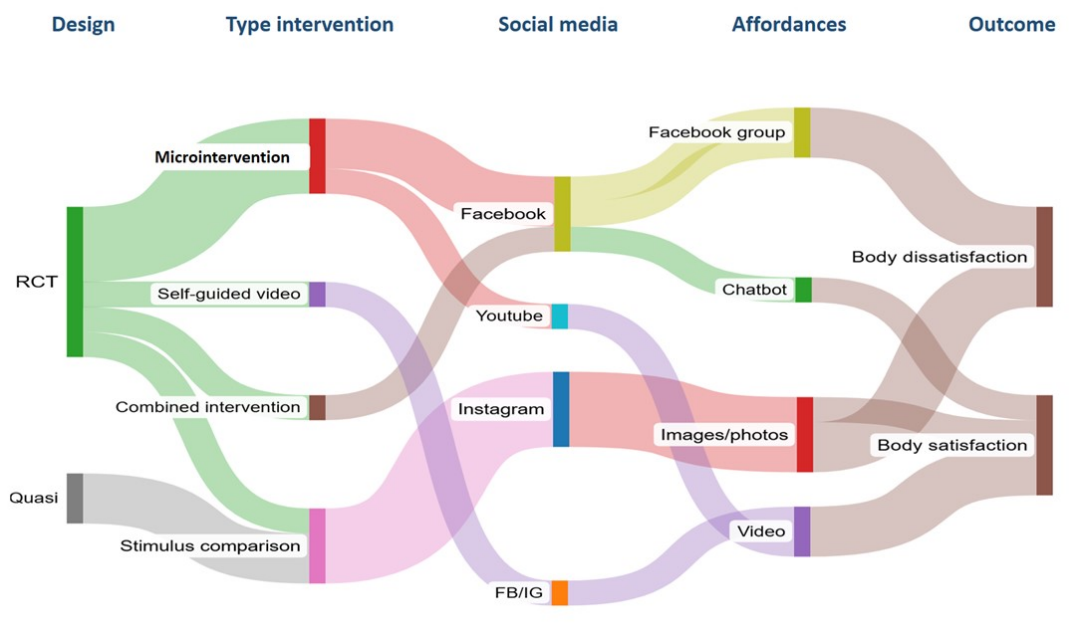
Graphical summary of the main characteristics and outcomes identified.

The total number of study participants across all studies was 4975 (approximately 17.7% boys [24,25] and 0.3% others [13]) with a sample size range from 76 to 1847 participants per study. The age range of participants was 13 to 30 years. There were 7 RCTs and 1 quasi-experimental study. Furthermore, 50% (4/8) of the studies included follow-up, ranging from 1 week to 3 months [24,36,40,41]. Control group conditions varied, including placebo (3/8, 42.8%) [25,37,39], waitlist (2/8, 28.6%) [40,41], and assessment-only control (2/8, 28.6%) [24,36] (Table 2; Figure 2). The instruments to assess BI were very diverse, including the visual analogical scale (n=3) [37-39], positive or negative affect (n=2) [24,41], and subscales of the eating disorders inventory (n=3) [36,39,40] (Table S1 in Multimedia Appendix 4) [24,25,36-41].

### The Effectiveness of Microintervention for Improving BI

Microinterventions, due to their ease of administration (ie, single or multiple), can generate an effective and positive sequence on the immediate impact of specific symptoms (eg, body satisfaction or dissatisfaction) [44]. The effectiveness of these interventions for improving BI is centered on reducing body comparison, developing strategies to mitigate criticism of media beauty standards, and cognitive reevaluation of body-related thoughts [45,46]. These types of interventions show positive effects when using gamified and interactive self-guided models or edutainment strategies for healthy behavior change [47,48]. Three studies included microinterventions to improve body satisfaction and/or dissatisfaction [24,36,37]. They offered easily understandable content to adolescents and young adults on a single occasion or over a specific period, the latter usually daily, with different degrees of success. For example, African American girls did not show any improvement in body satisfaction after watching content on social media for comparison and pressure in a drama miniseries format (*P*=.11) [37]. In another study, gamified chatbot-based models did improve the body self-esteem of boys and girls, although with a small effect size (*P*<.02) [24]. A third study exposed adolescents and young adults to body-positive content on Facebook in different presentations (eg, access to nonidealized social beauty content presented in photos, images, external links, or access to other social media) in specific Facebook groups, resulting in reduced body dissatisfaction (*P*=.03) and reduced tendency to compare appearance (*P*<.01) [36].

### The Effectiveness of Comparative Stimulus Interventions

Three studies assessed how specific social media features (eg, posting photos or images) can change some aspects of BI in adolescents and young adult women [25,38,39]. Exposure to idealized images (eg, attractive smiles of Instagram users) was found to reduce facial satisfaction (*P*<.01) [25]. In another study, creating profiles on social media platforms (eg, Instagram) with body-positive content increased positive mood and body satisfaction (ie, with the neutral image group) [38]. Strategies based on identifying self-affirming values ​(ie, selecting and reflecting on the most important value presented in the intervention) ​did not produce significant changes in BI and mood (*P*>.60) [39].

### The Effectiveness of a Combined Intervention

One study combined a workshop with group interactions on Facebook [40]. The brief workshop was focused on self-compassion competencies. Then, workshop participants shared previous workshop experiences and learnings in a private Facebook group. The evaluation showed a favorable reduction in body dissatisfaction (*P*<.01).

### The Effectiveness of Self-Guided Video Activities

One study used a self-guided educational model consisting of short, sequential videos. These videos were shared on social media (Facebook or Instagram) to explore BI risk factors among young Indonesian women [41]. These activities were effective in reducing body dissatisfaction in girls and young women (*P*<.01), although with a small effect size (η^2^=0.05).

### Efficacy of HVSM Interventions Comparison Between Adolescents and Young Adults

The studies targeting adolescents were mostly conducted in the Americas (ie, the United States or Brazil) with a variety of HVSM administration (eg, Facebook, Instagram, YouTube, or a combination of both HVSM) [24,37]. Overall, the time of the interventions in the adolescent group was shorter (ie, from 5 minutes to 3 days) compared to young adults (14 to 28 days) [24,25,36-40]. Only the adolescent group used video and chatbot interventions. Two of the 3 interventions for adolescents did improve BI, with small effects for reducing body dissatisfaction or improving body self-esteem (Cohen *d*=0.13) [24,41], while the interventions that achieved positive effects on BI in young adults focused on reducing body dissatisfaction.

### Risk of Bias

**Figure 3:**
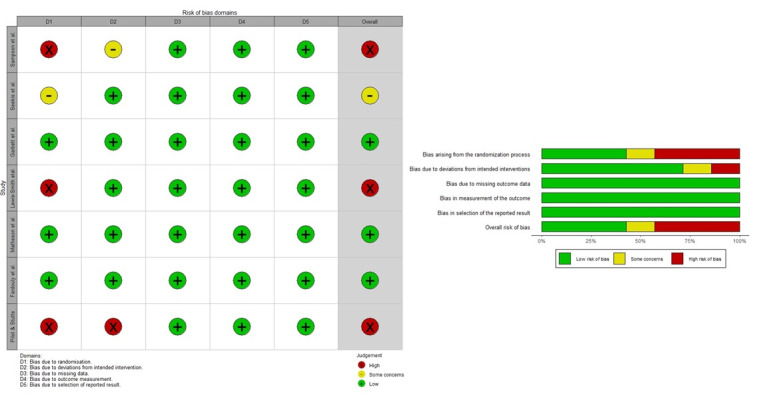
Risk of bias graph for randomized controlled trials.

A summary of the risk of bias assessment of the RCT studies is visible in Figure 3. Three studies did not report enough details on randomization [25,37,39,40], leading to internal validity uncertainty. One study raised concerns about bias resulting from potential overestimated intervention effects [25,39]. All studies presented low risk in terms of missing data, outcome measurement validity, and outcome selection [24,25,36,37,39,40,41].

## Discussion

### Principal Findings

This is the first systematic review of interventions that use HVSM platforms to tackle unhealthy BI in adolescents and young adults. While the number of available studies remains limited, the data suggest that these interventions—despite their variability—can support modest improvements in body-related outcomes. We classified these programs into 4 types: microinterventions, interventions based on comparative stimulus, combined interventions, and self-guided video activities. All intervention types showed improvements in BI, albeit with small effect sizes. Some risk of bias was detected, particularly a lack of randomization in half of the studies, which can compromise the validity of intervention effect estimates. Overall, the studies identified in the systematic review addressed both the increase in body satisfaction and the reduction in dissatisfaction.

Microinterventions can be effective under certain conditions, such as clarity in the actions directed to the target audience (eg, formative or interventionist), a combination of specific strategies (ie, how and when to implement the actions), including interactive strategies (eg, completing activities). Chatbot-based microintervention techniques (eg, social media content management, cognitive-behavioral models, and psychoeducational techniques) showed positive effects on body satisfaction [24]. Microinterventions using gamification for BI may be advantageous due to their narrow applicability (ie, even up to a single session), short duration (eg, videos less than 5 minutes long), and greater specificity (eg, adolescents in specific age ranges) [47,49]. Brief experiences followed by quick feedback—typical in social media—can produce rewarding experiences that promote engagement and adherence [50,51]. Similarly, microinterventions using a sequenced dosage of body-positive content (eg, photo posts, image posts, and links) in social media groups (eg, Facebook groups) were also beneficial in improving body satisfaction [36]. Thus, repeated and sequenced exposure to content that promotes body acceptance or is aesthetically neutral on a daily basis (eg, ~2 weeks and at least once a day) could reduce the internalization of beauty ideals and improve BI, acceptance, and body diversity by counteracting the effect of feeds of idealized images [52].

Exposure to positive content on social media could increase cognitive flexibility regarding the internalization of socially acceptable beauty standards [53]. Even viewing neutral content (eg, travel, landscapes, art, etc) can motivate young women to decentralize their BI (ie, interests beyond appearance) [54].

However, a microintervention based solely on a single online dramatization episode did not reduce body dissatisfaction [37]. This may be due to the indirect and unrealistic narrative (ie, presented in a single short video) of body dissatisfaction-related messages, as opposed to strategies based on successive videos (eg, miniseries or sequential videos) that can provide feedback on specific and direct topics [55]. The lack of human interaction may decrease the appeal of these interventions, especially for adolescents and young adults [12,47]. Likewise, having main characters that resemble thin-ideal standards (eg, in online series produced for an intervention) may encourage internalized appearance comparison, generating the indirect effect of body dissatisfaction [52].

Comparison stimuli based on positive images or photographs (eg, more realistic bodies) or neutral content (eg, nature, landscapes, or trips) can reduce the effects of body dissatisfaction [25,38,39]. Exposure to positive visual content can significantly reduce the evaluative determinants of body dissatisfaction (eg, increased stress and decreased subjective well-being) [56]. Neutral content (ie, related to nature) can generate subjective benefits through the sensation and appreciation of a healthy body [57,58]. However, images with thinness standards or fitspiration have been shown to increase thoughts directed toward social comparison with people subjectively perceived as superior (eg, artists, actors, influencers, or other stereotypes), increasing body dissatisfaction [59].

Exposure to sequential educational series (ie, more than one chapter) that included interactive activities (eg, completing a comic or identifying the message of a specific episode) reduced body dissatisfaction [41]. The use of entertainment series (eg, drama series designed with educational content) may have a positive effect on attitude change [60]. Thus, the familiarity that the characters can generate and the interaction with the quality of the storyline (ie, the anticipatory effect) play a crucial role in the incorporation of the desired messages about thought control and the elimination of personal comparisons that promote risk factors in body dissatisfaction [61].

The use of Facebook groups in combination with self-compassion workshops shows favorable results in reducing body dissatisfaction [40]. Combined strategies of conscious self-compassion through healthy self-talk, positive changes in self-worth, and physical warmth foster tolerance of one’s own physical appearance [62,63]. Added to the online component (eg, Facebook group), they can reduce appearance comparison ideals and body concerns. A combined intervention that includes active participation in Facebook groups (eg, reading others’ experiences and compassionate writing) may increase the benefits of joint feedback and how this can help recognize negative emotions related to corporality [64,65].

Despite the variability of the interventions, they share common elements such as (1) repeated dose and sequence (eg, microinterventions), (2) specific conceptual focus, that is, they must be centered on outcomes close to BI, and (3) adaptable, with plausible and culturally clear messages about body acceptance. Exposure to positive images on social media (eg, inspirational images that do not conform to hegemonic beauty standards) can foster self-improvement motives related to body satisfaction [38,66]. Furthermore, self-compassionate writing as a core component in interventions using private social media (eg, Facebook) can enhance interventions or workshops aimed at reducing body-related concerns [40,65]. Sequenced videos (eg, entertainment-education) primarily rely on psychoeducational components (eg, appearance-based comparisons) and elements of cognitive behavioral therapy, such as cognitive dissonance (ie, redirecting thoughts about physical changes), combined with interactive models that can generate motivation and adherence [24,41].

Our review identified differential effectiveness in BI interventions that align with the activities and needs of adolescents and young adults groups with a majority of women. In adolescent populations, interactive resources (eg, videos or game strategies) that incorporate cognitive-behavioral and coping components can help manage external validation, regulate feelings of BI self-worth, and address peer influence [24,41]. In contrast, young adults respond effectively to positive visual content (ie, photos, posts, or links), generating positive upward comparison (ie, motivation for self-improvement) [67]. Furthermore, the use of private groups can facilitate self-compassionate feedback to reduce preoccupation with rigid beauty ideals [65]. This suggests the need to develop differentiated strategies for both channels and content to optimize the effectiveness of interventions.

Overall, there is a methodological limitation in half of the studies, which may be considered unreliable results. Specifically, the studies by Sampson et al [25], Seekis et al [40], Pilot and Stutts [39], and Lewis-Smith et al [37] presented some risk of bias due to a lack of randomization and 2 studies also included issues regarding deviations from planned interventions [25,39]. Regardless of the intervention resource administered in HVSM, selection bias (D1) could exist, generating differences in potentially important prognostic characteristics (eg, related to baseline BI) [68,69]. This lack of comparability to overestimate the potential effects (eg, in a combined intervention workshop + Facebook group) means that the observed benefits could be attributed to the pre-existing characteristics of the participants, rather than the intervention itself [68]. Additionally, implementation bias (D2) could introduce further biases, such as potential contamination of the control group (eg, in interventions using HVSM), thus reducing the actual effects of the intervention group [69]. Despite this, another group of studies achieved a low risk of bias and identified high feasibility and acceptability with favorable results for HVSM interventions in different age groups (eg, adolescents and young adults) and intervention formats (ie, video, chat, and visual content) [24,36,41]. This suggests that platforms such as Facebook and Instagram (or others) could be useful for improving BI once the methodological limitations identified in this study are overcome, potentially providing a strong rationale for future interventions.

While other reviews have explored various digital interventions (eg, apps or websites), this research is innovative because it focuses exclusively on social media as a core component of interventions to improve BI. To our knowledge, this is the first review to synthesize evidence specifically on social media–based interventions for BI disturbance (rather than broader digital or eHealth approaches), thereby expanding our understanding of the potential use of social media as an intervention delivery channel and active therapeutic ingredient. This review extends prior work by moving beyond platform descriptions to examine how interventions operate, identifying putative mechanisms of action used regardless of platform (eg, social comparison, cognitive dissonance, self-compassion, and behavioral modeling) and the intervention format used (eg, video-based content, stimulus comparison, and microinterventions). The contributions to the field include evidence suggesting that brief interventions can still be effective and the potential for large-scale reach and scalability. Although studies of social media–based interventions remain limited, our findings highlight clear targets for optimization that future intervention developers can incorporate. In real-world terms, social media–based tools could be embedded within platforms or delivered alongside routine social media use to help prevent or reduce symptoms of BI disturbance and counterbalance exposure to unhealthy beauty standards among groups at higher risk, including adolescents [24,70]. Realizing this potential may require platform-level design changes, clearer safeguarding standards, and supportive policy and regulatory frameworks, alongside partnerships with civil society organizations working to protect young people’s mental health [15].

### Strengths and Limitations

This study offers the first systematic evaluation of BI interventions implemented through HVSM platforms among youth and emerging adults. Furthermore, our study identifies the content and digital affordances that can improve body satisfaction in this vulnerable population. However, in the search strategy, other terms that encompass the full meaning of BI may not have been captured. To mitigate this, the research team comprehensively mapped search terms that could potentially capture BI-related words. The small number of studies identified precluded a meta-analysis, which would help to provide cumulative evidence for informed recommendations and clinical decision-making. Nevertheless, our study is useful in summarizing the updated information on this type of cost-effective intervention with a high adherence rate in improving mental health problems.

### Conclusions

Interventions delivered through HVSM platforms to improve BI still require further development and research due to the preliminary evidence of their findings. Some formats—especially those that are interactive, brief, and visually engaging—appear to foster small but meaningful changes in how adolescents and young adults perceive their appearance. The identified methodological heterogeneity implies difficulty in obtaining a single estimate, which currently prevents a formal comparison of effects across included studies. This suggests that the interventions were designed exclusively for specific contexts and may not generate the same expected effects. However, this variety of intervention formats allows us to identify functional principles and adaptable conditions related to the dosage and mechanisms of healthy BI improvement, as well as the contextual sensitivity of success or failure in specific populations (eg, adolescents and young adults). Despite this challenge, social media remains a space with enormous reach and influence. If thoughtfully designed, interventions embedded in these platforms could become an important part of broader efforts to address BI issues and promote psychological well-being in vulnerable populations.

## Appendix

Multimedia Appendix 1 PRISMA checklist.

Multimedia Appendix 2 PRISMA-S checklist.

Multimedia Appendix 3 Search strategy.

Multimedia Appendix 4 Measurement tools.

## Abbreviations

BI: body image
HVSM: highly visual social media
MeSH: Medical Subject Headings
PRISMA: Preferred Reporting Items for Systematic Reviews and Meta-Analyses
PRISMA-S: Preferred Reporting Items for Systematic Reviews and Meta-Analyses Search Extension
RCT: randomized controlled trial

## Data Availability

The protocol generated or analyzed during this study is available in Zenodo [71].
