# Supplementary material for: Interventions That Use Highly Visual Social Media Platforms to Tackle Unhealthy Body Image in Adolescents and Young Adults: Systematic Review of Randomized Controlled Trials and Quasi-Experimental Studies
Source: J Med Internet Res. 2026 Feb 9;28:e80141. doi: 10.2196/80141 (PMC12930149; doi:10.2196/80141)
Supplement: Multimedia Appendix 2 [file jmir_v28i1e80141_app2.pdf]

PRISMA-S checklist

SECTION/TOPIC	# ITEM	CHECKLIST ITEM	Location where item is reported
**INFORMATION SOURCES AND METHODS**			
Database name	1	Name each individual database searched, stating the platform for each	7
Multi-database searching	2	If databases were searched simultaneously on a single platform, state the name of the platform, listing all of the databases searched.	7
Study registries	3	List any study registries searched.	7
Online resources and browsing	4	Describe any online or print source purposefully searched or browsed (e.g., tables of contents, print conference proceedings, web sites), and how this was done.	7
Citation searching	5	Indicate whether cited references or citing references were examined, and describe any methods used for locating cited/citing references (e.g., browsing reference lists, using a citation index, setting up email alerts for references citing included studies).	7
Contacts	6	Indicate whether additional studies or data were sought by contacting authors, experts, manufacturers, or others	7
Other methods	7	Describe any additional information sources or search methods used.	7
**SEARCH STRATEGIES**			
Full search strategies	8	Include the search strategies for each database and information source, copied and pasted exactly as run	8 and supplemental S2
Limits and restrictions	9	Specify that no limits were used, or describe any limits or restrictions applied to a search (e.g., date or time period, language, study design) and provide justification for their use.	8
Search filters	10	Indicate whether published search filters were used (as originally designed or modified), and if so, cite the filter(s) used.	8
Prior work	11	Indicate when search strategies from other literature reviews were adapted or reused for a substantive part or all of the search, citing the previous review(s).	8

Updates	12	Report the methods used to update the search(es) (e.g., rerunning searches, email alerts).	8
Dates of searches	13	For each search strategy, provide the date when the last search occurred.	9
**PEER REVIEW**			
Peer review	14	Describe any search peer review process.	8
**MANAGING RECORDS**			
Total records	15	Document the total number of records identified from each database and other information sources.	10
Deduplication	16	Describe the processes and any software used to deduplicate records from multiple database searches and other information sources.	8

From: http://dx.doi.org/10.1136/bmj.n160

PRISMA 2020 for Abstracts Checklist

Section and Topic	Item #	Checklist item	Reported (Yes/No)
**TITLE**			
Title	1	Identify the report as a systematic review.	Yes
**BACKGROUND**			
Objectives	2	Provide an explicit statement of the main objective(s) or question(s) the review addresses.	Yes
**METHODS**			
Eligibility criteria	3	Specify the inclusion and exclusion criteria for the review.	Yes
Information sources	4	Specify the information sources (e.g. databases, registers) used to identify studies and the date when each was last searched.	Yes
Risk of bias	5	Specify the methods used to assess risk of bias in the included studies.	yes
Synthesis of results	6	Specify the methods used to present and synthesise results.	yes
**RESULTS**			
Included studies	7	Give the total number of included studies and participants and summarise relevant characteristics of studies.	Yes
Synthesis of results	8	Present results for main outcomes, preferably indicating the number of included studies and participants for each. If meta-analysis was done, report the summary estimate and confidence/credible interval. If comparing groups, indicate the direction of the effect (i.e. which group is favoured).	Yes
**DISCUSSION**			
Limitations of evidence	9	Provide a brief summary of the limitations of the evidence included in the review (e.g. study risk of bias, inconsistency and imprecision).	Yes
Interpretation	10	Provide a general interpretation of the results and important implications.	Yes
**OTHER**			
Funding	11	Specify the primary source of funding for the review.	Yes
Registration	12	Provide the register name and registration number.	Yes

From: Page MJ, McKenzie JE, Bossuyt PM, Boutron I, Hoffmann TC, Mulrow CD, et al. The PRISMA 2020 statement: an updated guideline for reporting systematic reviews. BMJ 2021;372:n71. doi: 10.1136/bmj.n71. This work is licensed under CC BY 4.0. To view a copy of this license, visit https://creativecommons.org/licenses/by/4.0/
