# Supplementary material for: Interventions That Use Highly Visual Social Media Platforms to Tackle Unhealthy Body Image in Adolescents and Young Adults: Systematic Review of Randomized Controlled Trials and Quasi-Experimental Studies
Source: J Med Internet Res. 2026 Feb 9;28:e80141. doi: 10.2196/80141 (PMC12930149; doi:10.2196/80141)
Supplement: Multimedia Appendix 3 [file jmir_v28i1e80141_app3.docx]

**Multimedia Appendix 3**

Search strategy

| EMBASE |  |
| --- | --- |
| #1 | ('appearance'/exp OR appearance OR 'body attitude*' OR 'body checking'/exp OR 'body checking' OR 'body concern*' OR 'body esteem'/exp OR 'body esteem' OR 'body evaluation' OR 'body satisfaction'/exp OR 'body satisfaction' OR 'body dissatisfaction'/exp OR 'body dissatisfaction' OR 'body image'/exp OR 'body image' OR 'body functionality' OR 'body surveillance' OR 'body shame'/exp OR 'body shame' OR 'body positive'/exp OR 'body positive' OR 'body positivity'/exp OR 'body positivity' OR 'body acceptance' OR 'body pride' OR 'body preoccupation' OR 'body awareness'/exp OR 'body awareness' OR 'body anxiety' OR 'body appreciation'/exp OR 'body appreciation' OR 'body neutrality' OR 'physical self perception' OR 'physical self-perception' OR 'physical self concept' OR 'physical self-concept' OR 'shape concern*' OR 'weight concern*' OR 'shape satisfaction' OR 'shape dissatisfaction' OR interocep* OR 'weight dissatisfaction' OR 'weight satisfaction' OR 'body ideal*' OR 'appearance ideal*' OR 'muscularity'/exp OR muscularity OR 'thin ideal' OR 'physical appearance'/exp OR 'physical appearance' OR muscular* OR 'social physique anxiety'/exp OR 'social physique anxiety' OR 'self objectification'/exp OR 'self objectification' OR 'objectified body consciousness' OR 'thinness'/exp OR thinness OR 'cognitive restraint'/exp OR 'cognitive restraint' OR 'body identity' OR 'body schema'/exp OR 'body schema' OR 'body representation*' OR 'self-esteem'/exp OR 'self-esteem' OR 'eating disorders':kw,ab,ti) AND ('article'/it OR 'clinical trial'/it) |
| #2 | (internet OR 'social media' OR 'social network site*' OR instagram OR facebook OR 'tik tok' OR 'tiktok' OR twitter OR reddit OR telegram OR whapsapp OR weibo OR snapchat OR youtube OR pinterest:kw,ti,ab) AND ('article'/it OR 'clinical trial'/it) |
| #3 | (child* OR adolescent* OR young OR youth OR teen* OR college OR universit* OR 'young adult*':ab,kw,ti) AND ('article'/it OR 'clinical trial'/it) |
| #4 | (intervention* OR 'controlled trial' OR 'clinical trial' OR 'major clinical trial' OR 'quasi-experimental' OR 'quasi experimental':ab,kw,ti) AND ('article'/it OR 'clinical trial'/it) |
| #5 | #1 AND #2 AND #3 AND #4 AND [2012-2025]/py |
| SCOPUS |  |
| #1 | TITLE-ABS-KEY(appearance OR "body anxiety" OR "body attitude*" OR "body checking" OR "body concern*" OR "body esteem" OR "body evaluation" OR "body satisfaction" OR "body dissatisfaction" OR "body image" OR "body functionality" OR "body surveillance" OR "body shame" OR "body positive" OR "body positivity" OR "body acceptance" OR "body pride" OR "body preoccupation" OR "body awareness" OR "body anxiety" OR "body appreciation" OR "body neutrality" OR "physical self perception" OR "physical self-perception" OR "physical self concept" OR "physical self-concept" OR "shape concern*" OR "weight concern*" OR "shape satisfaction" OR "shape dissatisfaction" OR interocep* OR "weight dissatisfaction" OR "weight satisfaction" OR "body ideal*" OR "appearance ideal*" OR muscularity OR "thin ideal" OR "physical appearance" OR muscular* OR "social physique anxiety" OR "self objectification" OR self-objectification OR "objectified body consciousness" OR thinness OR "cognitive restraint" OR "body identity" OR "body schema" OR "body representation*" OR "self-esteem" OR "eating disorders") |
| #2 | TITLE-ABS-KEY(internet OR "social media" OR "social network site*" OR instagram OR facebook OR "tik tok" OR "TikTok" OR twitter OR reddit OR telegram OR whapsapp OR weibo OR snapchat OR youtube OR pinterest) |
| #3 | TITLE-ABS-KEY(Child* OR adolescent* OR young OR youth OR teen* OR college OR universit* OR "Young Adult*") |
| #4 | TITLE-ABS-KEY(intervention* OR "controlled trial" OR "clinical trial" OR "major clinical trial" OR "quasi-experimental" OR "quasi experimental") |
| #5 | #1 AND #2 AND #3 AND #4 AND PUBYEAR > 2011 AND PUBYEAR < 2026 |
| MEDLINE |  |
| #1 | (appearance or "body anxiety" or "body attitude*" or "body checking" or "body concern*" or "body esteem" or "body evaluation" or "body satisfaction" or "body dissatisfaction" or "body image" or "body functionality" or "body surveillance" or "body shame" or "body positive" or "body positivity" or "body acceptance" or "body pride" or "body preoccupation" or "body awareness" or "body anxiety" or "body appreciation" or "body neutrality" or "physical self perception" or "physical self-perception" or "physical self concept" or "physical self-concept" or "shape concern*" or "weight concern*" or "shape satisfaction" or "shape dissatisfaction" or interocep* or "weight dissatisfaction" or "weight satisfaction" or "body ideal*" or "appearance ideal*" or muscularity or "thin ideal" or "physical appearance" or muscular* or "social physique anxiety" or "self objectification" or self-objectification or "objectified body consciousness" or thinness or "cognitive restraint" or "body identity" or "body schema" or "body representation*" or "self-esteem" or "eating disorders").ab,kw,ti. |
| #2 | (internet or "social media" or "social network site*" or instagram or facebook or "tik tok" or "TikTok" or twitter or reddit or telegram or whapsapp or weibo or snapchat or youtube or pinterest).ab,kw,ti. |
| #3 | (Child* or adolescent* or young or youth or teen* or college or universit* or "Young Adult*").ab,kw,ti. |
| #4 | (intervention* or "controlled trial" or "clinical trial" or "major clinical trial" or "quasi-experimental" or "quasi experimental").ab,kw,ti. |
| #5 | #1 AND #2 AND #3 AND #4 |
| #6 | limit 5 to yr="2012 - 2025" |
| WOS |  |
| #1 | appearance OR "body anxiety" OR "body attitude*" OR "body checking" OR "body concern*" OR "body esteem" OR "body evaluation" OR "body satisfaction" OR "body dissatisfaction" OR "body image" OR "body functionality" OR "body surveillance" OR "body shame" OR "body positive" OR "body positivity" OR "body acceptance" OR "body pride" OR "body preoccupation" OR "body awareness" OR "body anxiety" OR "body appreciation" OR "body neutrality" OR "physical self perception" OR "physical self-perception" OR "physical self concept" OR "physical self-concept" OR "shape concern*" OR "weight concern*" OR "shape satisfaction" OR "shape dissatisfaction" OR interocep* OR "weight dissatisfaction" OR "weight satisfaction" OR "body ideal*" OR "appearance ideal*" OR muscularity OR "thin ideal" OR "physical appearance" OR muscular* OR "social physique anxiety" OR "self objectification" OR self-objectification OR "objectified body consciousness" OR thinness OR "cognitive restraint" OR "body identity" OR "body schema" OR "body representation*" OR "self-esteem" OR "eating disorders" (Topic) |
| #2 | internet OR "social media" OR "social network site*" OR instagram OR facebook OR "tik tok" OR "TikTok" OR twitter OR reddit OR telegram OR whapsapp OR weibo OR snapchat OR youtube OR pinterest (Topic) |
| #3 | Child* OR adolescent* OR young OR youth OR teen* OR college OR universit* OR "Young Adult*" (Topic) |
| #4 | intervention* OR "controlled trial" OR "clinical trial" OR "major clinical trial" OR "quasi-experimental" OR "quasi experimental" (Topic) |
| #5 | #1 AND #2 AND #3 AND #4 |
| APAPsyinfo |  |
| #1 | (appearance or "body anxiety" or "body attitude*" or "body checking" or "body concern*" or "body esteem" or "body evaluation" or "body satisfaction" or "body dissatisfaction" or "body image" or "body functionality" or "body surveillance" or "body shame" or "body positive" or "body positivity" or "body acceptance" or "body pride" or "body preoccupation" or "body awareness" or "body anxiety" or "body appreciation" or "body neutrality" or "physical self perception" or "physical self-perception" or "physical self concept" or "physical self-concept" or "shape concern*" or "weight concern*" or "shape satisfaction" or "shape dissatisfaction" or interocep* or "weight dissatisfaction" or "weight satisfaction" or "body ideal*" or "appearance ideal*" or muscularity or "thin ideal" or "physical appearance" or muscular* or "social physique anxiety" or "self objectification" or self-objectification or "objectified body consciousness" or thinness or "cognitive restraint" or "body identity" or "body schema" or "body representation*" or "self-esteem" or "eating disorders").mp. [mp=title, abstract, heading word, table of contents, key concepts, original title, tests & measures, mesh word] |
| #2 | (internet or "social media" or "social network site*" or instagram or facebook or "tik tok" or "TikTok" or twitter or reddit or telegram or whapsapp or weibo or snapchat or youtube or pinterest).mp. [mp=title, abstract, heading word, table of contents, key concepts, original title, tests & measures, mesh word] |
| #3 | Child* or adolescent* or young or youth or teen* or college or universit* or "Young Adult*").mp. [mp=title, abstract, heading word, table of contents, key concepts, original title, tests & measures, mesh word] |
| #4 | (intervention* or "controlled trial" or "clinical trial" or "major clinical trial" or "quasi-experimental" or "quasi experimental").mp. [mp=title, abstract, heading word, table of contents, key concepts, original title, tests & measures, mesh word] |
| #5 | 1 and 2 and 3 and 4 |
