# Supplementary material for: Interventions That Use Highly Visual Social Media Platforms to Tackle Unhealthy Body Image in Adolescents and Young Adults: Systematic Review of Randomized Controlled Trials and Quasi-Experimental Studies
Source: J Med Internet Res. 2026 Feb 9;28:e80141. doi: 10.2196/80141 (PMC12930149; doi:10.2196/80141)
Supplement: Multimedia Appendix 4 [file jmir_v28i1e80141_app4.docx]

**Multimedia Appendix 4**

**Measurements Tools**

Wide range of variables related to body image resulted in heterogeneity among the different assessment instruments. Various self-report measures (e.g. Likert scales) were assessed on mental health outcomes mainly related to body image. The majority of studies (n = 7; 41.2%) assessed outcomes related to appearance and weight, self-esteem and sociocultural attitudes. 29.4% (n = 5) of the studies measured outcomes that were directly related to body image (i.e. body appreciation, body image self-efficacy, facial satisfaction, etc.). Moreover, all five studies (29.4%) included negative body image outcomes (e.g. eating disorder, appearance comparison or weight-related eating) (see table 3). Furthermore, in most studies a visual analogue scale (VAS) was used as a complement to self-reports. However, only one study presents two psychometric properties (validity and reliability) [45] (see table S1)

**Table S1.** Psychometric quality of the reported measures for body image.

| psychometric quality | author (year) | measures |
| --- | --- | --- |
| Weak* | Sampson et al (2020) [25] | BSS; FSS; SACS; SDI |
| Moderate** | Fiorivanti et al (2023) [43] | C-VAS; SACS; PACS; MBSRQ-AS |
| Moderate** | Garbett et al (2023) [46] | BESAA; SATAQ-3; PANAS-C |
| Moderate** | Pilot et al (2023) [44] | Sub-scale EDEQ; WREQ; VAS |
| Moderate** | Lewis-Smith et al (2023) [42] | VAS; sub-scale IROS |
| Moderate** | Matheson et al (2023) [24] | BESAA; PANAS; BISES |
| Moderate** | Fardouly et al (2023) [41] | Sub-scale EDI; BAS-2; PANAS-SF; SOBBS; PACS; SAM modified |
| Strong*** | Seekis et al (2020) [45] | Sub-scale EDI; SAAS; UPACS; BAS-2; SCS-SF |

Note. The research team assessed the quality of the validity and reliability information for the scales used in all studies as ***strong (reported validity and reliability), **moderate (reported validity or reliability), and *weak (no validity and reliability reports). BSS Body Satisfaction Scale; FSS Facial Satisfaction Scale; SDI Self-Discrepancy Index; EDI-3 Eating Disorder Inventory; EDI-3 Eating Disorder Inventory (Drive for Thinness Subscale); SAAS Social Appearance Anxiety Scale; UPACS Upward Physical Appearance Comparison Scale; SCS-SF Self-Compassion Scale–Short-Form; C-VAS Computer-based Visual Analogue Scale; SACS State Appearance Comparison Scale; MBSRQ-AS Multidimensional Body-Self Relations Questionnaire Appearance Scales; PANAS-C Positive and Negative Affect Schedule for Children; SATAQ-3 Sociocultural Attitudes Towards Appearance Questionnaire; Eating Disorder Examination Questionnaire (weight/shape subscales); WREQ Weight-related eating questionnaire; BESAA Body Esteem Scale for Adolescents and Adults; VAS Visual Analogue Scales; IROS Internalized Racial Oppression Scale (Alteration of Physical Appearance and Hair Change subscales); BISES Body Image Self-Efficacy Scale; EDI Eating Disorder Inventory (only body weight and shape); BAS-2 Body Appreciation Scale-2; PANAS-SF Positive and Negative Affect Scale-Short Form; SOBBS Self-Objectification Beliefs and Behaviors Scale; PACS Physical Appearance Comparison Scale; SAM modified version of the social activism measure.

Our findings identified the use of different tools to assess positive or negative body image. [25,35,41,45,46,46] These factors assessed multidimensional and, in some cases, unidimensional measures (i.e., they only took one factor), which could compromise the general understanding of the evaluated construct [70]. However, the presentation of the instruments was complemented by the use of other scales that evaluated physical appearance related to emotional factors and the use of visual analogue scales, which could improve an evaluation from two fronts: a) the detailed understanding of the experience of the construct and b) a rapid assessment of its intensity [71], which can guarantee a broad information of the variables evaluate.
